# Supplementary material for: The OTUD6B‐LIN28B‐MYC axis determines the proliferative state in multiple myeloma
Source: EMBO J. 2022 Sep 5;41(20):e110871. doi: 10.15252/embj.2022110871 (PMC9574752; doi:10.15252/embj.2022110871)
Supplement: Supplementary file 1 — Appendix [file EMBJ-41-e110871-s006.pdf]

Appendix Table of Content

Appendix Table S1……………………………………………………Page 2

Appendix Figure S1……………………………………………………Page 4

Appendix Figure S2……………………………………………………Page 5

Appendix Figure S3……………………………………………………Page 6

Appendix Figure S4……………………………………………………Page 7

Appendix Figure S5……………………………………………………Page 8

Appendix Table S1

Tagreted Gene	Average Scored	sgRNA	individual sgRNA score
NonTargetingControlGuideForHuman_0001			1.35782647
NonTargetingControlGuideForHuman_0002			0.9982045
NonTargetingControlGuideForHuman_0003			0.63068416
NonTargetingControlGuideForHuman_0004			1.43014221
NonTargetingControlGuideForHuman_0005			0.95797421
NonTargetingControlGuideForHuman_0006			0.91375329
NonTargetingControlGuideForHuman_0007			1.71145245
NonTargetingControlGuideForHuman_0008			1.44506761
NonTargetingControlGuideForHuman_0009			1.66801323
NonTargetingControlGuideForHuman_0010			1.12208588
NonTargetingControlGuideForHuman_0011			1.63814176
NonTargetingControlGuideForHuman_0012			1.18684872
POLR2I	0.158868986	sgRNA_1	0.12570087
		sgRNA_2	0.09578823
		sgRNA_3	0.25511786
PRPF8	0.783241797	sgRNA_1	0.45331051
		sgRNA_2	0.19606252
		sgRNA_3	1.70035236
RPA3	0.157340769	sgRNA_1	0.21480297
		sgRNA_2	0.14524138
		sgRNA_3	0.11197796
RPL32	0.543137945	sgRNA_1	0.05338584
		sgRNA_2	1.4771775
		sgRNA_3	0.09885049
RPL8	0.119488394	sgRNA_1	0.13367376
		sgRNA_2	0.18636555
		sgRNA_3	0.03842587
RPS19	0.572376108	sgRNA_1	1.11685611
		sgRNA_2	0.52316681
		sgRNA_3	0.0771054
USP2	0.796991185	sgRNA_1	0.98250212
		sgRNA_2	0.67557247
		sgRNA_3	0.73289897
OTUD6B	0.786228859	sgRNA_1	1.15605302
		sgRNA_2	0.54523303
		sgRNA_3	0.65740053
MYSM1	0.769617137	sgRNA_1	0.8915798
		sgRNA_2	0.6880567
		sgRNA_3	0.72921491
USP5	0.751379878	sgRNA_1	0.50927813
		sgRNA_2	1.36193941
		sgRNA_3	0.3829221
USP12	0.518587398	sgRNA_1	0.64686387
		sgRNA_2	0.438321
		sgRNA_3	0.47057732
PSMD14	0.515694287	sgRNA_1	0.08544051
		sgRNA_2	0.32041492
		sgRNA_3	1.14122743
COPS5	0.471614476	sgRNA_1	1.07595112
		sgRNA_2	0.01200759
		sgRNA_3	0.32688472
BAP1	0.453885878	sgRNA_1	0.42546899
		sgRNA_2	0.61878554
		sgRNA_3	0.31740311
USP17L5	0.382964456	sgRNA_1	0.25129263
		sgRNA_2	0.10958025
		sgRNA_3	0.78802049
USPL1	0.209391892	sgRNA_1	0.14787642
		sgRNA_2	0.22018881
		sgRNA_3	0.26011045
USP17L11	0.154930978	sgRNA_1	0.06742691
		sgRNA_2	0.2681702
		sgRNA_3	0.12919583

Non-targeting control
Essential gene
DUB scroing < 0.8
OTUD6B

Appendix Table S1: sgRNA representation of a DUB CRISPR drop out screen in MM1.S cells. Non-targeting control sgRNAs (green), essential genes as control (red) and DUBs, which show a fold change below 0.8 at day 14 (orange), are listed. The ratio of normalized sgRNA read-counts on day 14 versus day 0 was determined for each sgRNA. For essential genes and DUBs the fold changes are depicted as average per gene and for each single sgRNA per gene. OTUD6B is marked in a darker shade of orange.

Appendix Figures

Appendix Figure S1

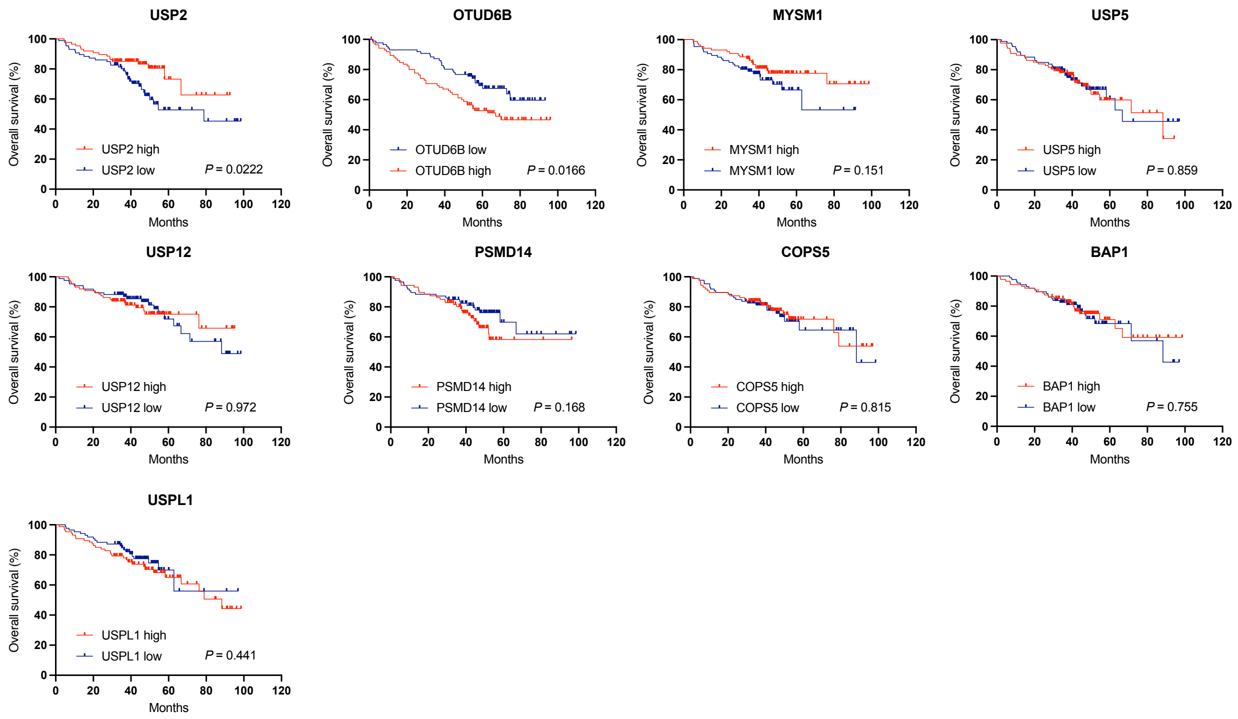

Appendix Figure S1. Kaplan-Meier survival curves of MM patients with high or low expression of different DUBs that scored in the CRISPR-dropout screen described in Figure 1A and Fig. EV1. Patients from the GSE24080 dataset were analyzed for their expression of a DUB of interest and the ones from the upper and lower quartiles were taken for analysis (n=173). *P* values calculated by Log-rank test. A more detailed analysis for OTUD6B can be found in Figure 6C, D.

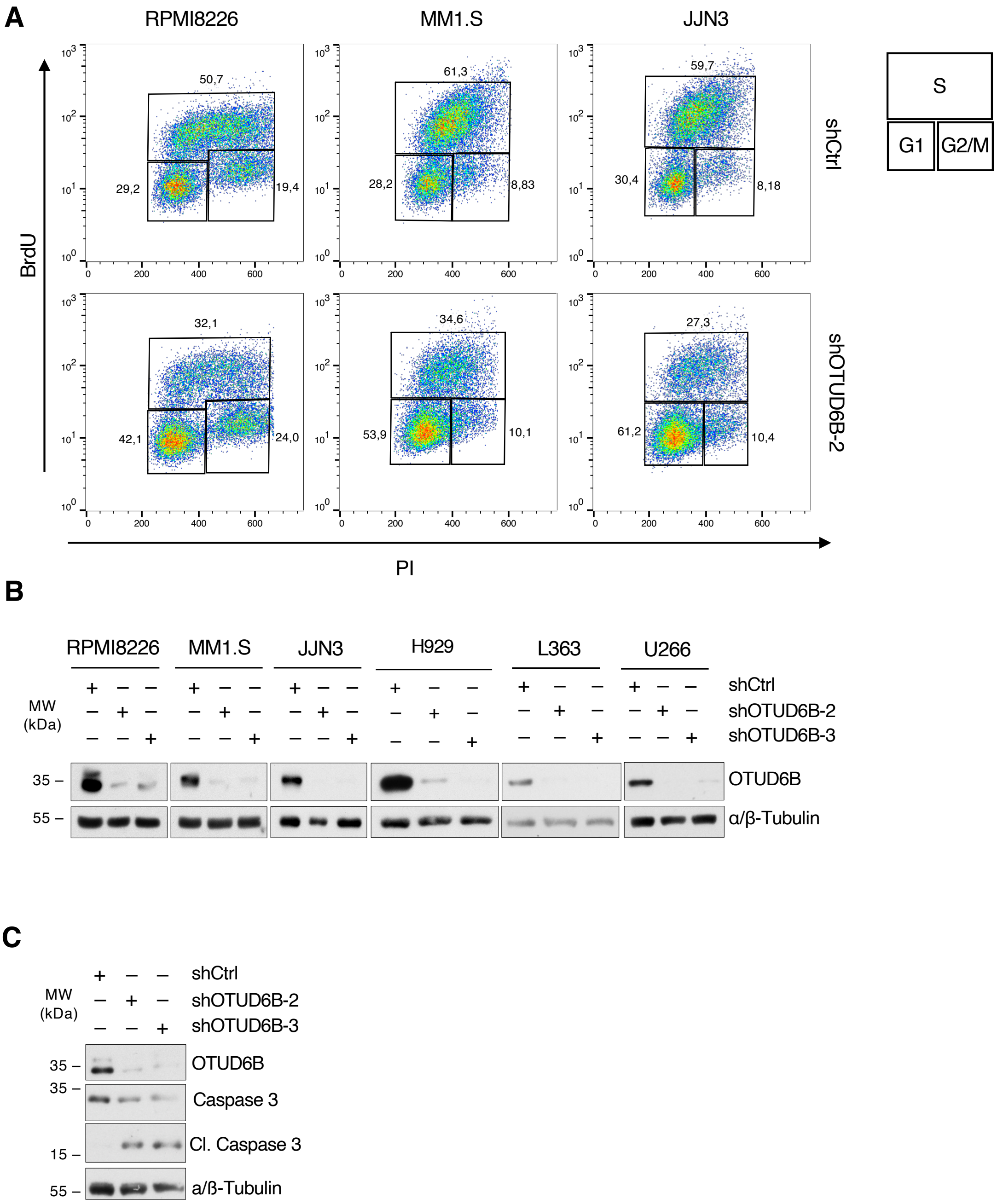

Appendix Figure S2. OTUD6B drives the G1/S cell cycle transition.
A, Two-dimensional cell cycle analysis of different MM cell lines expressing the indicated shRNAs analysed by BrdU/PI staining. Data are depicted as graphs of BrdU positive cells versus PI/DNA staining. **B**, Representative immunoblot analysis of cells described in **Fig. 1B and C** using the indicated antibodies. **C**, Immunoblot analysis of RPMI8226 cells infected with the indicated shRNAs. Cells were collected 8 days after infection and WCE analysed using the indicated antibodies. a/ß-Tubulin was used as loading control.

Appendix Figure S3

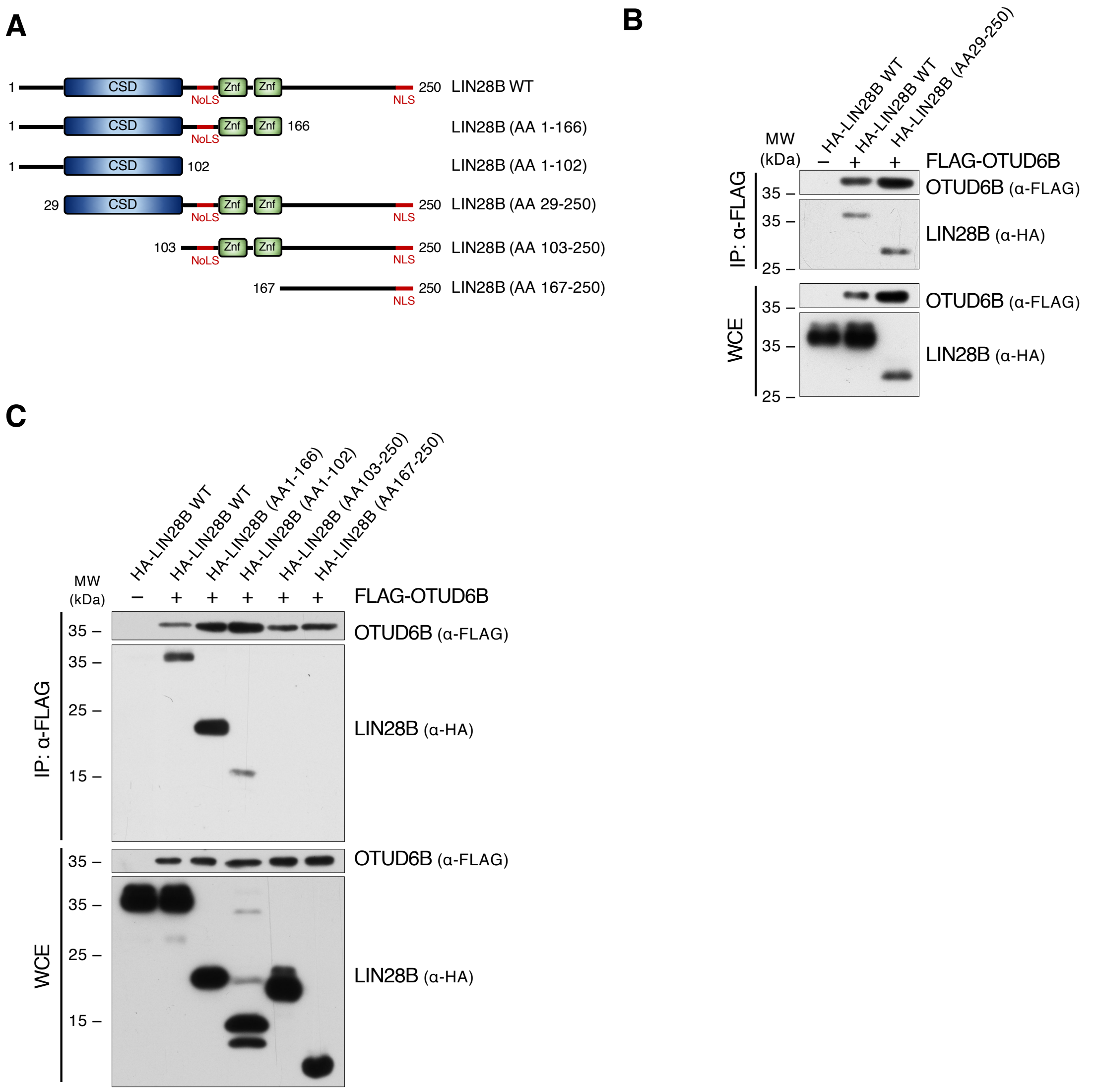

Appendix Figure S3. OTUD6B interacts with the cold shock domain of LIN28B.
A, Schematic overview of LIN28B wildtype (WT) and different deletion mutants thereof. AA, amino acid; CSD, cold shock domain; NLS, nuclear localization sequence; NoLS, nucleolar localization sequence; ZnF, zinc finger. **B, C**, Interaction analysis of FLAG-OTUD6B with HA-tagged LIN28B fragments. FLAG-OTUD6B and different HA-tagged fragments of LIN28B depicted in (**A**) were co-expressed in HEK293T cells and WCE subjected to FLAG-IP. Results were analysed by immunoblot using the indicated antibodies.

Appendix Figure S4

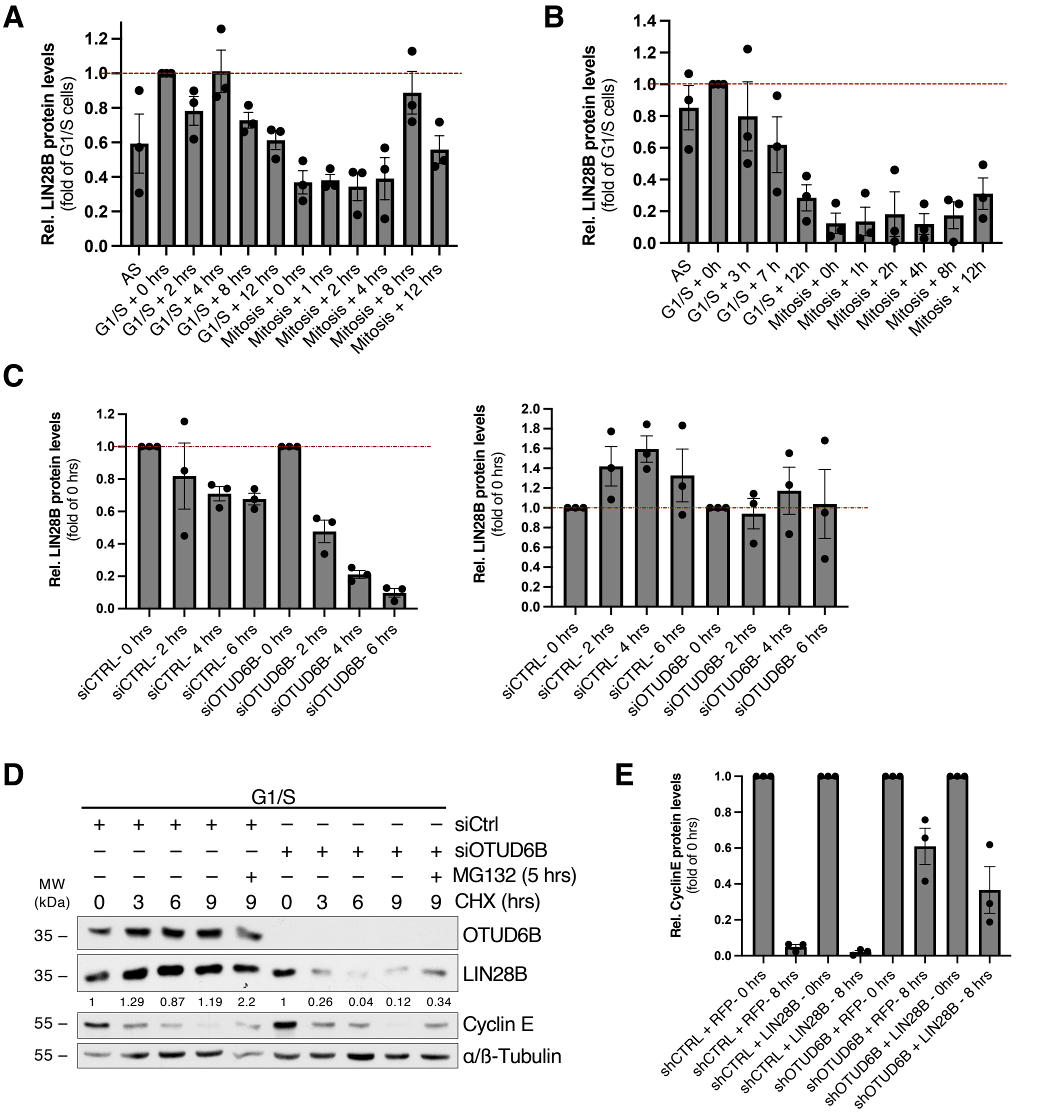

Appendix Figure S4. OTUD6B stabilizes LIN28B at the G1/S transition.
A, B, Quantification of LIN28B protein levels throughout the cell cycle in (**A**) RPMI8226 and (**B**) A549 cells from three independent experiments as depicted in Figure 3B, C. Values are normalized to G1/S arrested cells. **C**, Quantification of LIN28B protein levels upon different durations of Cycloheximide (CHX) addition in G1/S-arrested (left) and asynchronous (right) A549 cells upon OTUD6B depletion as described in Figure 3D from three independent experiments. Values are normalized to 0 hrs of treatment. **D,** Immunoblot analysis of LIN28B protein half-life in G1/S-synchronized A549 cells upon OTUD6B depletion. Cells transfected with the respective siRNAs were synchronized in G1/S-phase or not and treated with CHX and MG132 as indicated. WCE were analyzed by immunoblotting using the indicated antibodies. Numbers indicate the relative LIN28B levels. **E,** Quantification of Cyclin E protein levels from three independent experiments depicted in Figure 4D. Values are depicted as fold of 0 hrs after G1/S release. Data Information: **A-E** Optical densities were measured using the LICORlite image studio software. Values are normalized to the presented loading control and depicted as mean ± s.d.

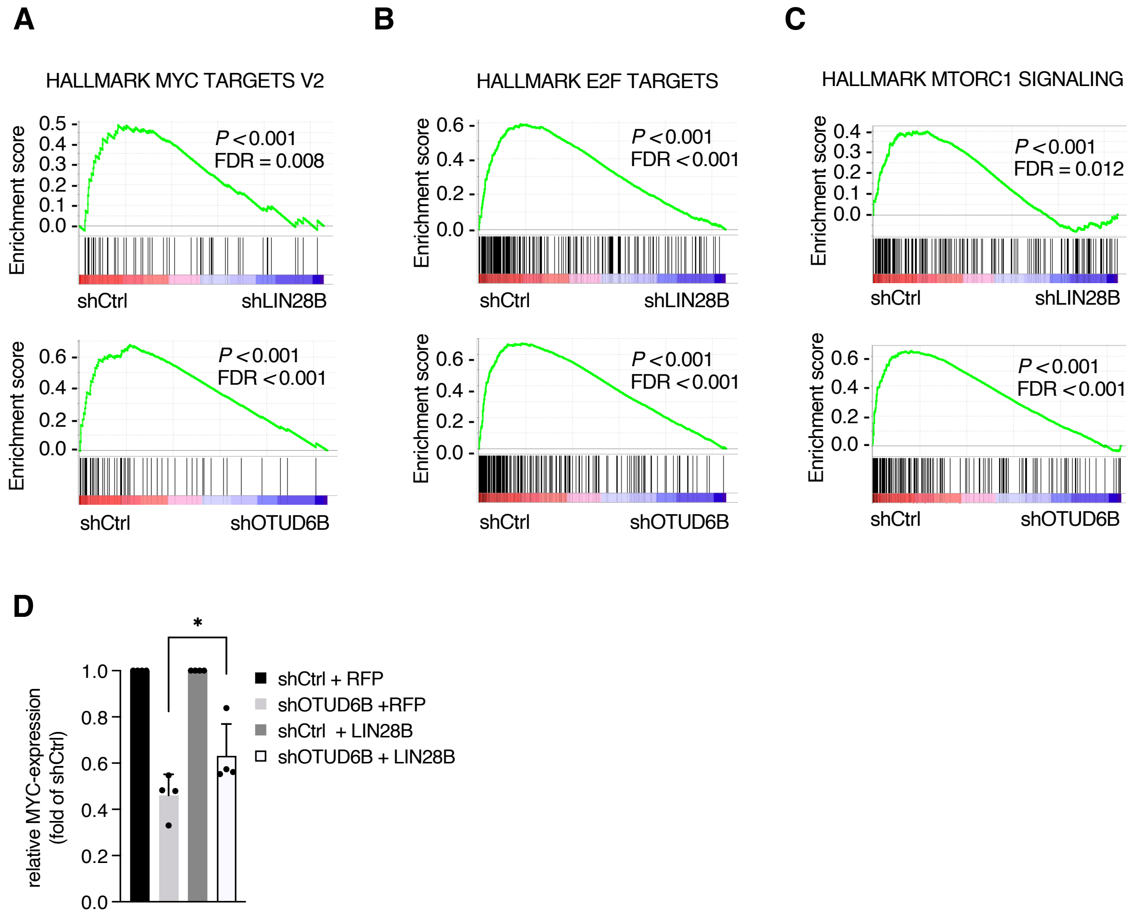

Appendix Figure S5. OTUD6B regulates central LIN28B target genes including MYC.
A-C, GSEAs showing an enrichment of MYC V2 **(A)**, E2F **(B)** and MTORC1 **(C)** hallmark target genes in *OTUD6B* and *LIN28B* depleted cells as compared to control cells. RPMI8226 cells expressing the indicated shRNAs were subjected to RNA-seq analysis and analysed for target gene expression **D,** Real-time qPCR of RPMI8226 cells expressing the indicated shRNAs and doxycycline-inducible constructs of either RFP (EV) or LIN28B. RPMI8226 cells stably expressing doxycycline inducible RFP or LIN28B were transduced with either shCtrl or shOTUD6B, transgene expression induced by doxycycline addition (1µg/mL, 1 day after infection) and samples collected three days post infection. Values are normalized to shCtrl. (n = 4 independent experiments with three technical replicates each); Values represent mean ± s.d. *, P < 0.05; by Student's t-test.
