# Supplementary material for: The OTUD6B‐LIN28B‐MYC axis determines the proliferative state in multiple myeloma
Source: EMBO J. 2022 Sep 5;41(20):e110871. doi: 10.15252/embj.2022110871 (PMC9574752; doi:10.15252/embj.2022110871)
Supplement: Supplementary file 2 — Expanded View Figures PDF [file EMBJ-41-e110871-s001.pdf]

Expanded View Figures

Figure EV1. A CRISPR screen for DUB vulnerabilities in MM identifies OTUD6B.

A Schematic depiction of the DUB-CRISPR drop out screen workflow.

B Proliferation analysis of MM1.S-Cas9 cells infected with indicated sgRNAs. The ratio of sgRNA expressing (GFP^+) cells to uninfected cells was measured by flow cytometry at the indicated time points after infection. Results are normalized to GFP^+ cells at day 4 (n = 3 independent experiments, mean ± s.d.).

C Immunoblot analysis verifying OTUD6B knockout in Cas9 expressing MM1.S cells infected with the indicated sgRNAs. GFP^+ cells were sorted by FACS and harvested 5 days after infection. Whole cell extracts (WCE) were probed with the indicated antibodies. CUL1 served as a loading control.

D Proliferation analysis of three additional Cas9 expressing MM cell lines lentivirally transduced with the indicated sgRNAs. The ratio of sgRNA expressing (GFP^+) to uninfected cells was measured by flow cytometry at the indicated time points after infection. Results are normalized to day 4 after infection (n = 4 independent experiments, mean ± s.d.).

E Immunoblot analysis verifying OTUD6B knockout in Cas9 expressing MM cell lines, which were transduced with indicated sgRNAs. GFP^+ cells were sorted by FACS 11 days after infection and WCE probed with indicated antibodies.

F Proliferation analysis of RPMI8226 cells stably expressing OTUD6B isoform 1 from a doxycycline inducible promotor and the indicated shRNAs with or without doxycycline mediated transgene induction. Cells were transduced with pTRIPZ-OTUD6B-IF1 and selected for vector expression using puromycin. Next, cells were infected as indicated with shCtrl or shOTUD6B-3, which binds in the 3'UTR of the gene, and either left untreated (DMSO) or OTUD6B-transgene expression induced by doxycycline addition (DOX, 1 μg/ml) the day after infection. Cell numbers were assessed by trypan-blue exclusion method 4 days after infection. Cell numbers are depicted as fold of DMSO with mean ± s.d from three independent experiments.

G Representative immunoblot analysis of WCE from cells collected at the time of proliferation analysis described in (F) using the indicated antibodies.

Data information: P-values *$P < 0.05$; **$P < 0.01$; by paired Student's t-test.
Source data are available online for this figure.

 © 2022 The Authors

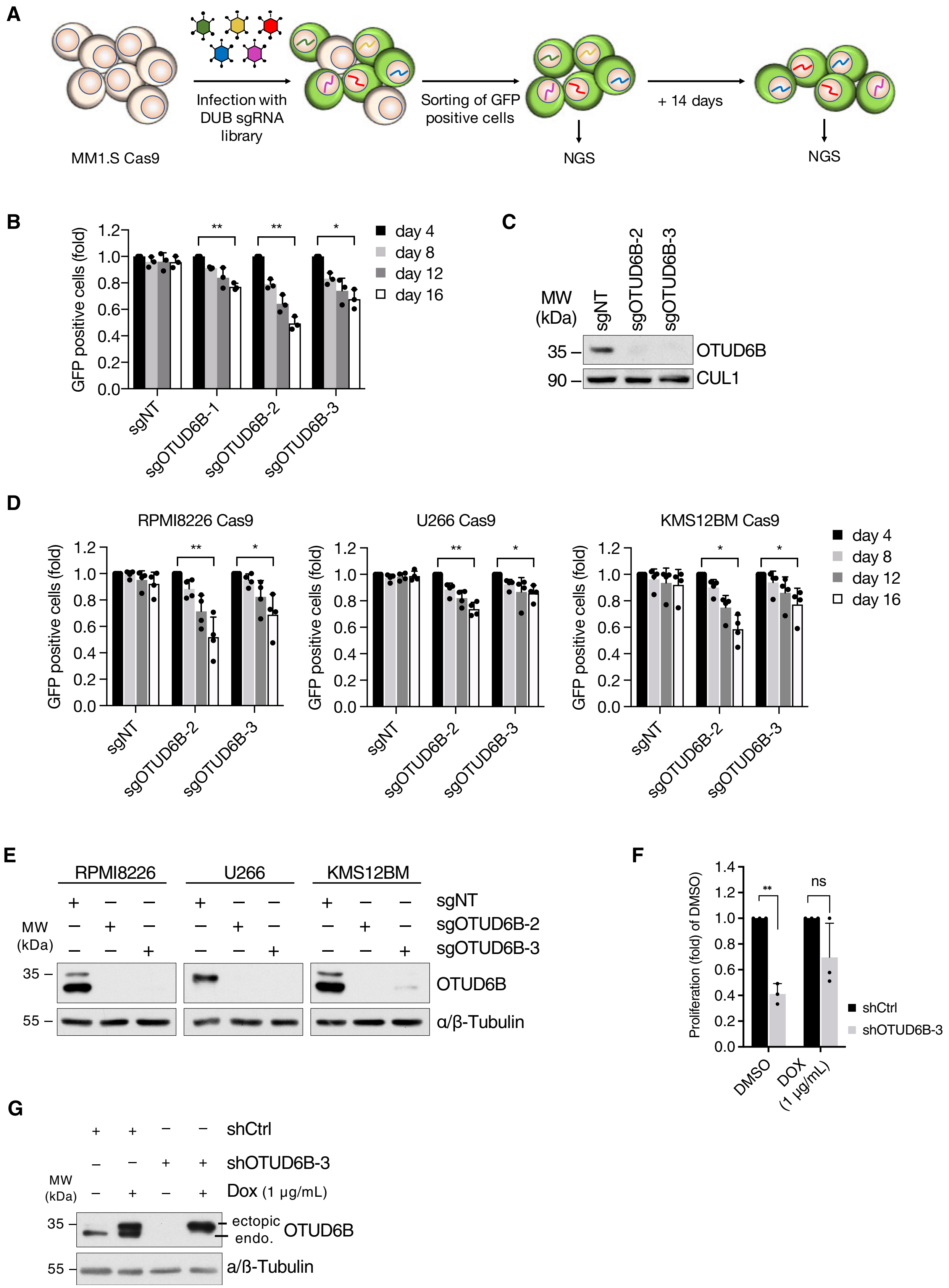
A
MM1.S Cas9
Infection with DUB sgRNA library
Sorting of GFP positive cells
+ 14 days
NGS
NGS
B
GFP positive cells (fold)
sgNT
sgOTUD6B-1
sgOTUD6B-2
sgOTUD6B-3
day 4
day 8
day 12
day 16
C
MW (kDa)
sgNT
sgOTUD6B-2
sgOTUD6B-3
OTUD6B
CUL1
D
RPMI8226 Cas9
U266 Cas9
KMS12BM Cas9
E
RPMI8226
U266
KMS12BM
sgNT
sgOTUD6B-2
sgOTUD6B-3
OTUD6B
α/β-Tubulin
F
Proliferation (fold) of DMSO
shCtrl
shOTUD6B-3
DMSO
DOX (1 µg/mL)
ns
G
shCtrl
shOTUD6B-3
Dox (1 µg/mL)
ectopic
endo.
OTUD6B
a/ß-Tubulin

Figure EV1.

Figure EV2. OTUD6B is a vulnerability with cell cycle dependent activity in both MM and LUAC cells.

A Proliferation analysis of A549-Cas9 cells infected with indicated sgRNAs. The ratio of sgRNA expressing (GFP^+) to uninfected cells was measured by flow cytometry at the indicated time points after infection. Results are normalized to GFP^+ cells at day 4 (n = 3 independent experiments).

B Two-dimensional cell cycle analysis of A549 lung adenocarcinoma cells expressing the indicated shRNAs analysed by BrdU/PI staining. Data from one representative experiment are depicted as graphs of BrdU positive cells versus PI/DNA staining.

C Quantitative cell cycle analysis of A549 cells expressing the indicated shRNAs for 5 days by BrdU/PI flow cytometry as depicted in (B) (n = 3 independent experiments).

D Quantification of OTUD6B activity from three independent experiments as depicted in Fig 1F. Optical densities were measured using the LICOR image studio lite software. Values are depicted as fold of OTUD6B-signal in G1/S arrested cells from three independent experiments.

E DUB activity assay for OTUD6B in asynchronous, G1/S- or mitotically synchronized RPMI8226 cells using HA-ubiquitin vinyl-sulfone to isolate active forms of DUBs followed by HA-IP. Analysis was performed by immunoblotting using the indicated antibodies.

Data information: Data in (A, C, D) are mean ± s.d. *$P < 0.05$; **, ***$P < 0.001$, $P < 0.01$; ****$P < 0.0001$; by paired Student's t-test corrected for multiple testing (C). Source data are available online for this figure.

 © 2022 The Authors

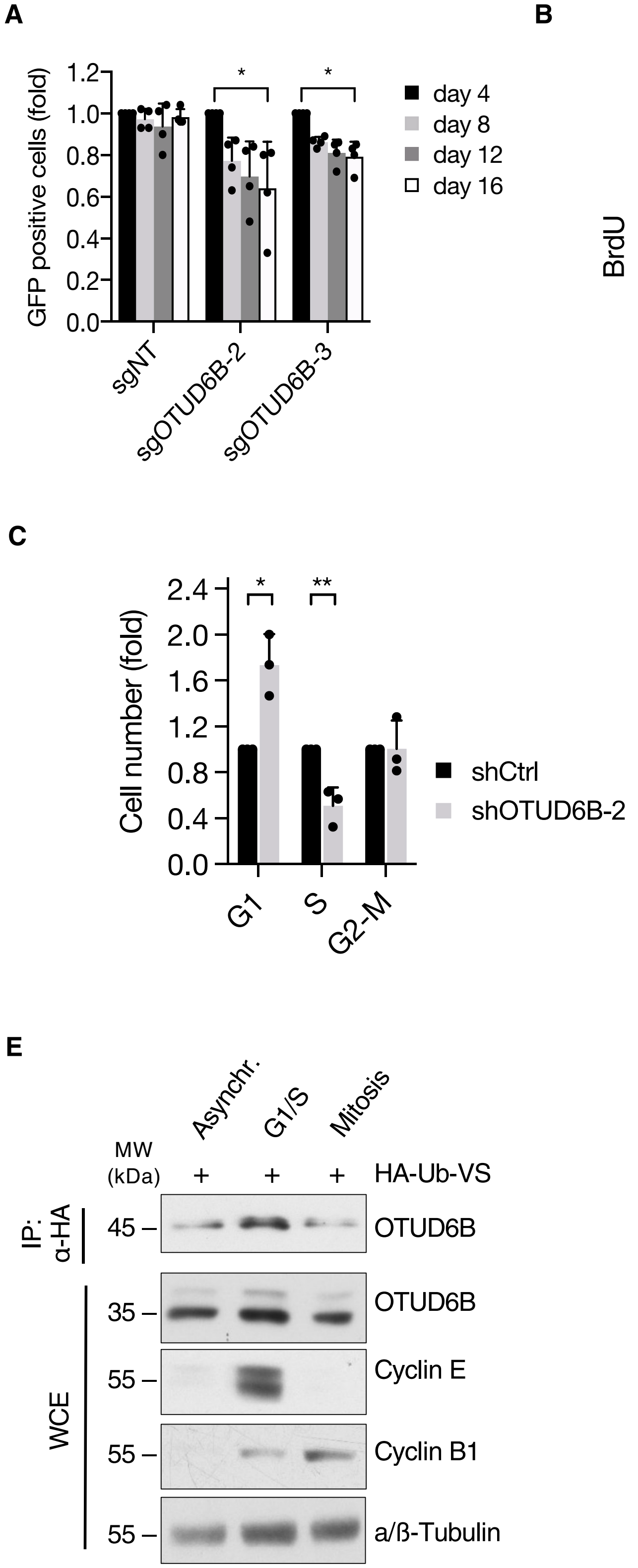
A
GFP positive cells (fold)
1.2
1.0
0.8
0.6
0.4
0.2
0.0
*
*
day 4
day 8
day 12
day 16
sgNT
sgOTUD6B-2
sgOTUD6B-3
B
shCtrl
shOTUD6B-2
BrdU
PI
40,0
51,5
7,94
13,0
75,5
10,2
C
Cell number (fold)
2.4
2.0
1.6
1.2
0.8
0.4
0.0
*
**
shCtrl
shOTUD6B-2
G1
S
G2-M
D
relative OTUD6B activity
(fold of G1/S cells)
1.6
1.2
0.8
0.4
0.0
****
***
*
asynchr.
G1/S 0 hrs
G1/S 6 hrs
G2/M 0 hrs
G2/M 4 hrs
E
Asynchr.
G1/S
Mitosis
MW
(kDa)
+ + +
HA-Ub-VS
IP:
α-HA
45 –
OTUD6B
WCE
35 –
OTUD6B
55 –
Cyclin E
55 –
Cyclin B1
55 –
a/ß-Tubulin

Figure EV2.

Figure EV3. Different Proteome-wide screening approaches identify LIN28B as an interaction partner of OTUD6B.

A Silver-stain analysis of FLAG-purified OTUD6B. HEK293T were transfected with either FLAG-EV or FLAG-OTUD6B isoform 1 (IF1). FLAG-OTUD6B was immunoprecipitated from WCE using FLAG M2 beads and subsequently eluted by 3x FLAG peptide. 1% of the purification was separated by SDS-PAGE and proteins visualized by silver staining. The arrowhead points to the band at the expected size of OTUD6B.

B Silver-stained gel of BioID2 purified proteins. HEK293T cells were transfected with MYC-tagged-BioID2 alone or BioID2-OTUD6B-isoform-1 (IF1) or -2 (IF2) fusion proteins, incubated with 50 μM biotin for 16 h and biotinylated proteins purified for MS-analysis. Non-transfected cells served as a control. The arrowheads point to the bands at the expected sizes of BioID2 alone, BioID2-OTUD6B-IF1 and -IF2.

C Results of mass spectrometric (MS) based screening for OTUD6B substrates correlating the results from the FLAG- and the Bio-ID-purification (A, B) as shown in Fig 2A. Intensities (LFQs) of co-immunoprecipitated proteins identified by MS were log2 transformed and the differences between OTUD6B and EV analyzed in both purifications. Proteins identified in both FLAG-IP-samples and enriched more than two-fold in both independent screens are listed. OTUD6B (bait) is marked in red and LIN28B in orange.

D Co-Immunoprecipitation (IP) of FLAG-tagged DUBs and endogenous LIN28B from HEK293T cells. IPs and WCE were analyzed by immunoblotting with the indicated antibodies.

E Co-IP of endogenous OTUD6B with FLAG-LIN28B.

F Co-IP of endogenous LIN28B with FLAG-OTUD6B-IF1 and FLAG-OTUD6B-IF2.

Data information: In (D–F) FLAG-tagged proteins were expressed in HEK293T cells, WCE subjected to FLAG-IP and both analysed by immunoblot using the indicated antibodies.

Source data are available online for this figure.

 © 2022 The Authors

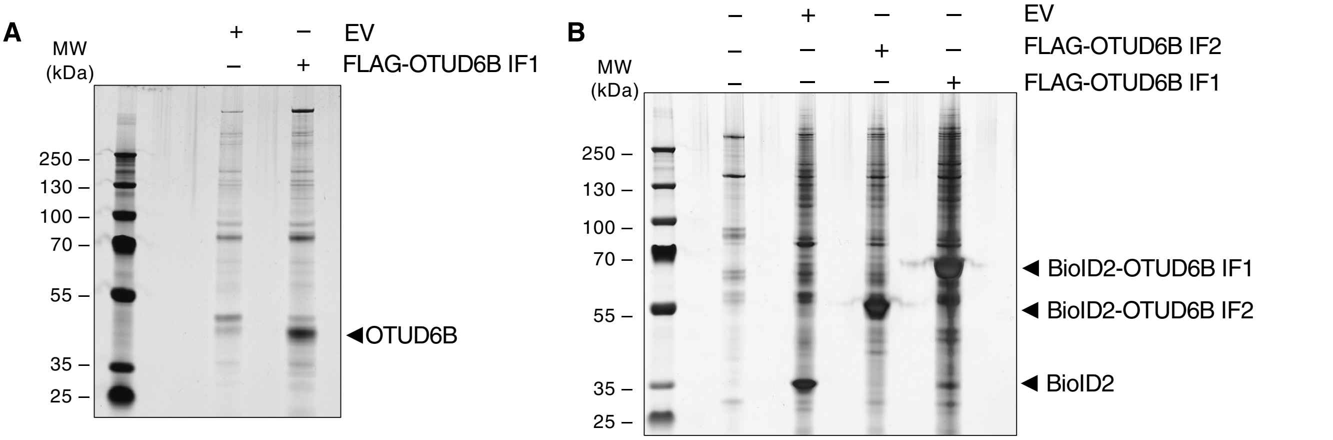

C

Gene names	OTUD6B -FLAG-purification log2(LFQ_Intensity OTUD6B/LFQ_Intesnity EV)	OTUD6B -BIO-ID log2(LFQ_Intensity OTUD6B/LFQ_Intesnity EV)
DNAJC10	4.407624089	1.192329147
SERBP1	3.804865222	1.316957217
LIN28B	3.799402459	3.687258528
DHX57	3.454191882	1.789527285
OTUD6B	3.327792717	9.825465328
RPL29	2.891893902	1.309078104
RPS15	2.859367794	2.903010096
FAU	2.841827464	2.364433193
RPL6	2.783159921	2.519741017
RPS11	2.732550304	1.212002936
STAU1	2.702199773	1.684860405
RPS8	2.699383694	2.260094592
DHX29	2.61130473	1.397181958
KNOP1	2.595290973	1.265515521
RPS18	2.586970427	1.292975593
RPL36	2.546093341	1.641315939
RPL4	2.475996785	1.19984861
RPL5	2.419130789	3.665721166
RPL18	2.355878029	5.361986881
RPL10A	2.309324205	1.450846546
RPL7A	2.294103818	1.119839273
PURA	2.281825215	14.26722561
RPL19	2.213216144	3.047475414
RPL14	2.165894028	1.305920935
YBX1	2.164546735	1.455279026
LTV1	2.157504064	1.917428243
H1FX	2.155840051	1.889175581
NCL	2.151464674	1.523438785
RPL7	2.136086024	1.101685509
CASC3	2.114607601	1.165177405
RPLP0;RPLP0P6	2.098218715	3.755246439
RPL32	2.034823015	1.599618179
RPL35A	1.96493378	3.384173093

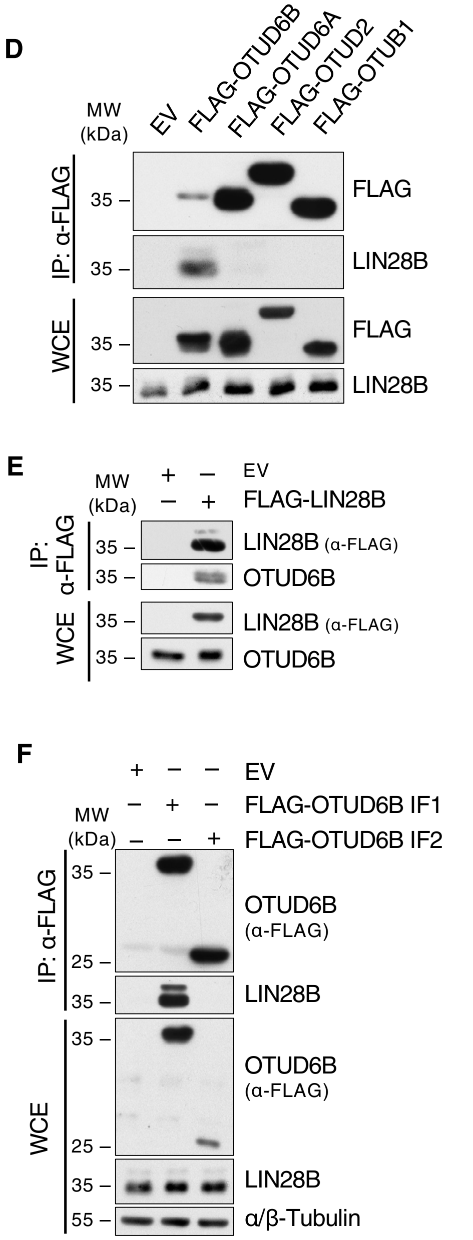

Figure EV3.

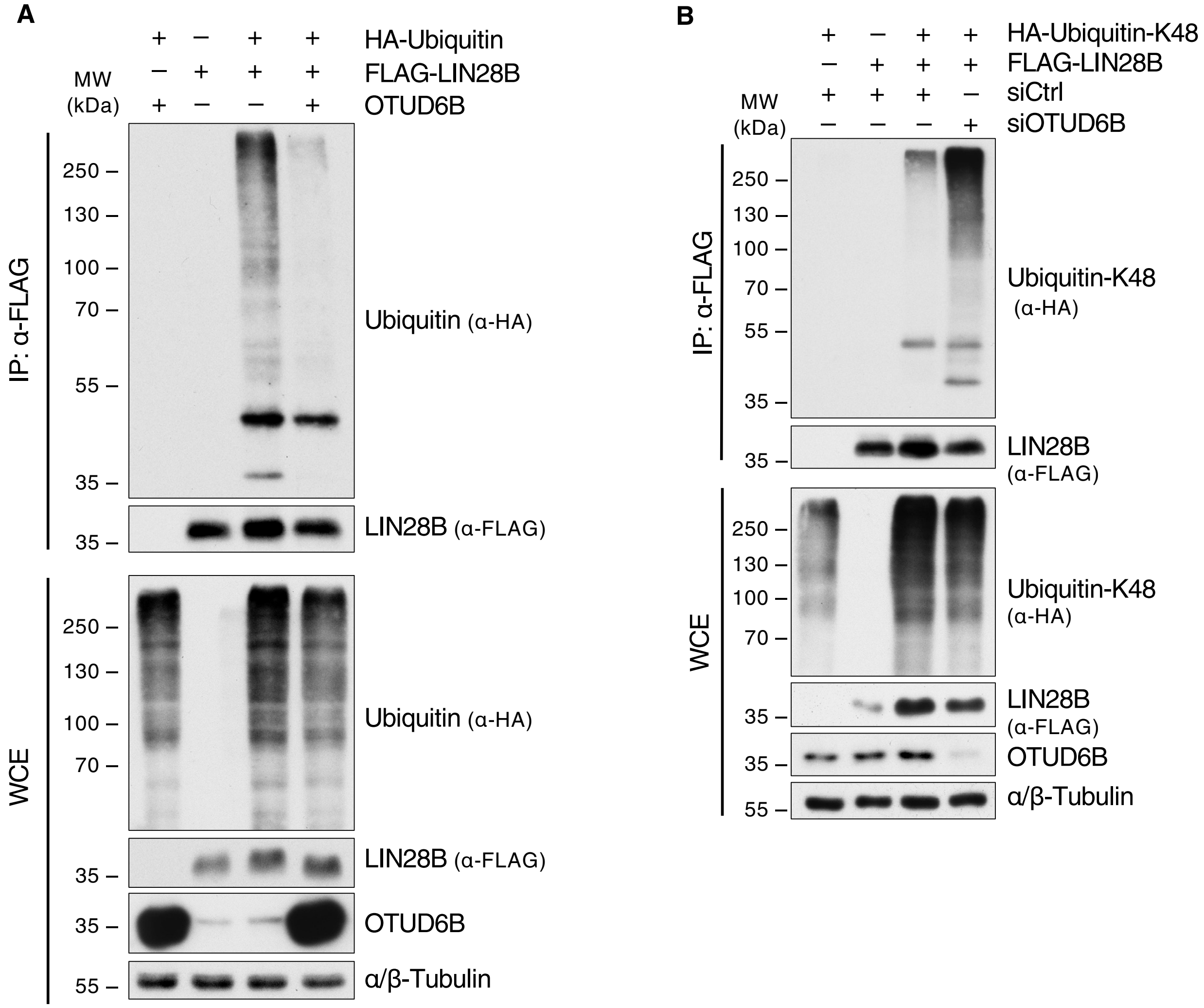

Figure EV4. LIN28B is a K48-specific de-ubiquitylation substrate of OTUD6B.

A *In vivo* ubiquitylation assay of LIN28B in OTUD6B overexpressing cells. HEK293T cells were transfected with indicated combinations of FLAG-LIN28B, HA-Ubiquitin, OTUD6B and EV control and treated with MG132 for 3 h 24 h later. Denatured WCE were subjected to FLAG-IP. WCE and IP were analysed by immunoblotting.

B *In-vivo* ubiquitylation analyses of LIN28B in HEK293T cells in which OTUD6B was silenced by siRNA using a K48-only-ubiquitin-mutant. Cells were transfected with the indicated siRNAs and overexpression constructs, then treated with MG132 for 3 h. Lysis and IP was done under denaturing conditions followed by WB analysis.

Source data are available online for this figure.

 © 2022 The Authors

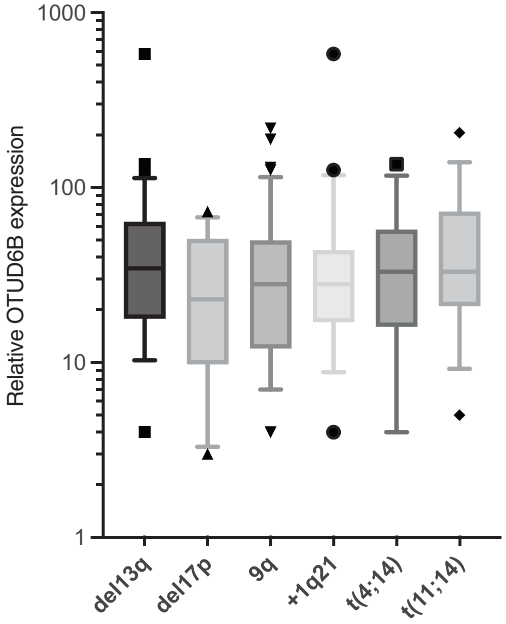

Figure EV5. OTUD6B levels do not correlate with a specific cytogenetic abnormality in MM patients.

Correlation between clinically predictive cytogenetic abnormalities and OTUD6B mRNA expression in MM patient samples at diagnosis. mRNA expression in primary $CD138^+$ MM cells was quantified by real-time qPCR (n = 89 patients from Fig 6A) and correlated with routinely assessed cytogentic profiles. Box-and-whisker plots show the upper and lower quartiles (25–75%) with a line at the median, whiskers extend from the 10th to the 90th percentile, and dots correspond values outside the 10th and 90th percentile.

Source data are available online for this figure.
