# Supplementary figures and images for: The OTUD6B‐LIN28B‐MYC axis determines the proliferative state in multiple myeloma
Source: EMBO J. 2022 Sep 5;41(20):e110871. doi: 10.15252/embj.2022110871 (PMC9574752; doi:10.15252/embj.2022110871)

**Fig. 1E**

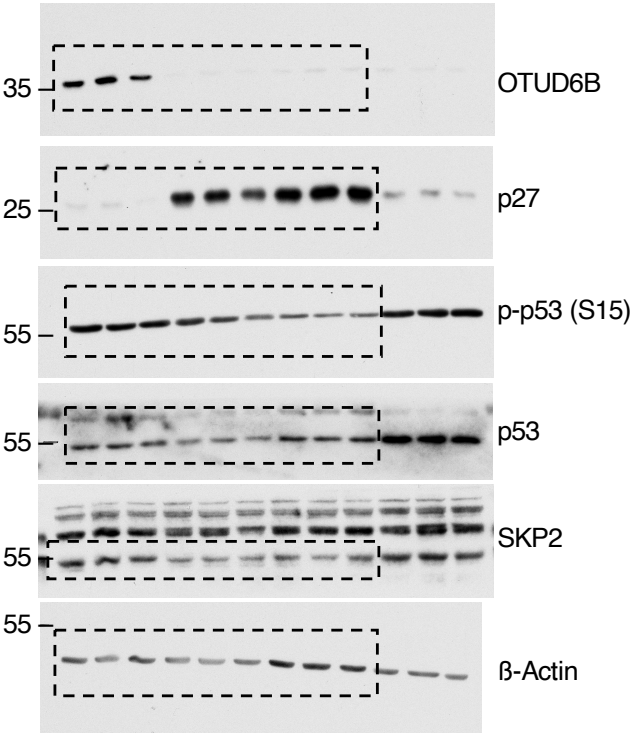

**Fig. 1F**

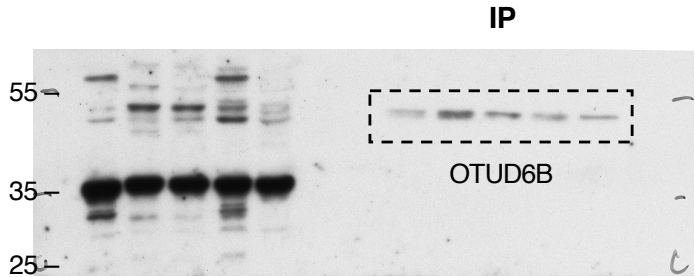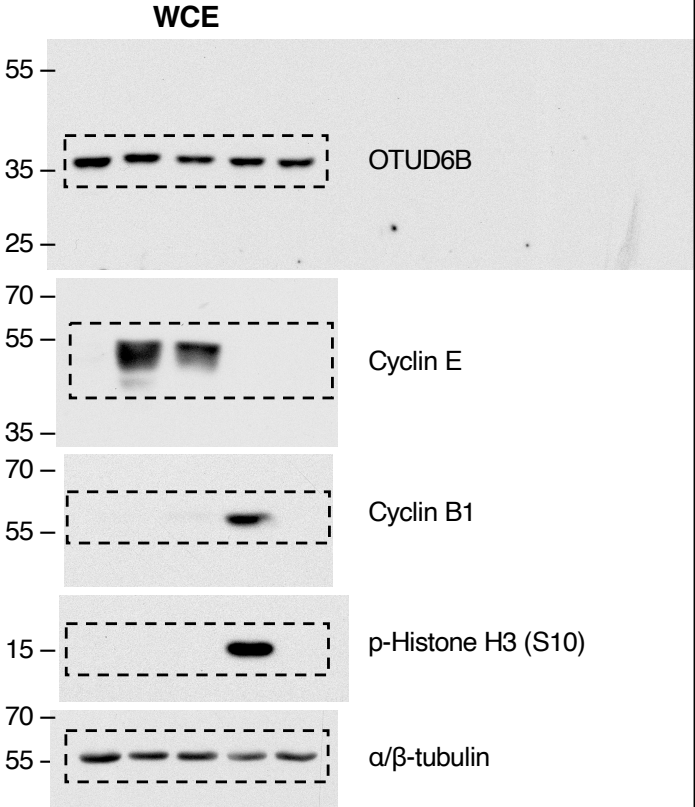

Supplement: Supplementary file 6 — Source Data for Figure 1 [file EMBJ-41-e110871-s002.zip › Source Data Fig. 1_uncropped.pdf]

**Fig. 2B**

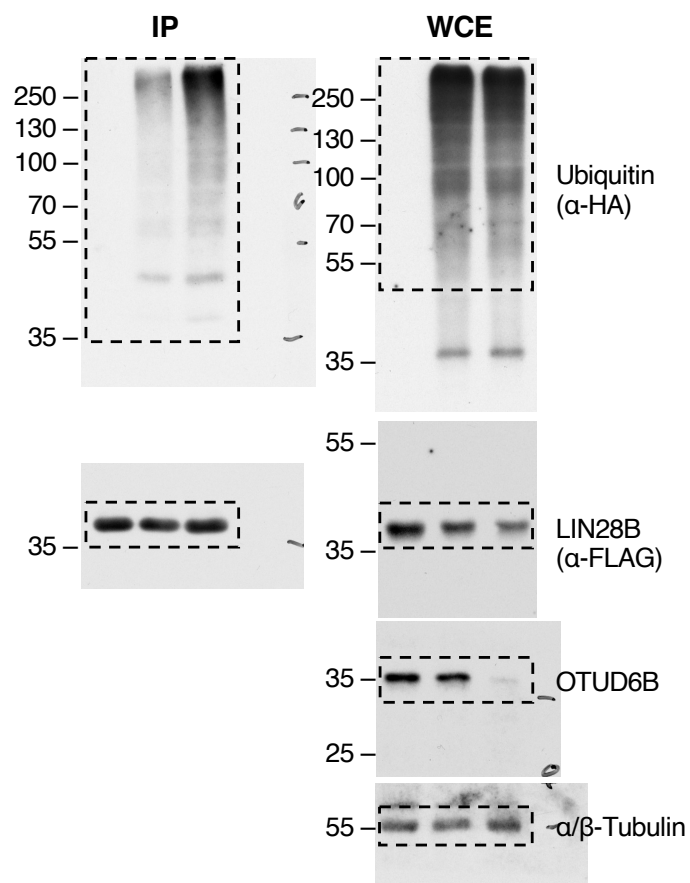

**Fig. 2C**

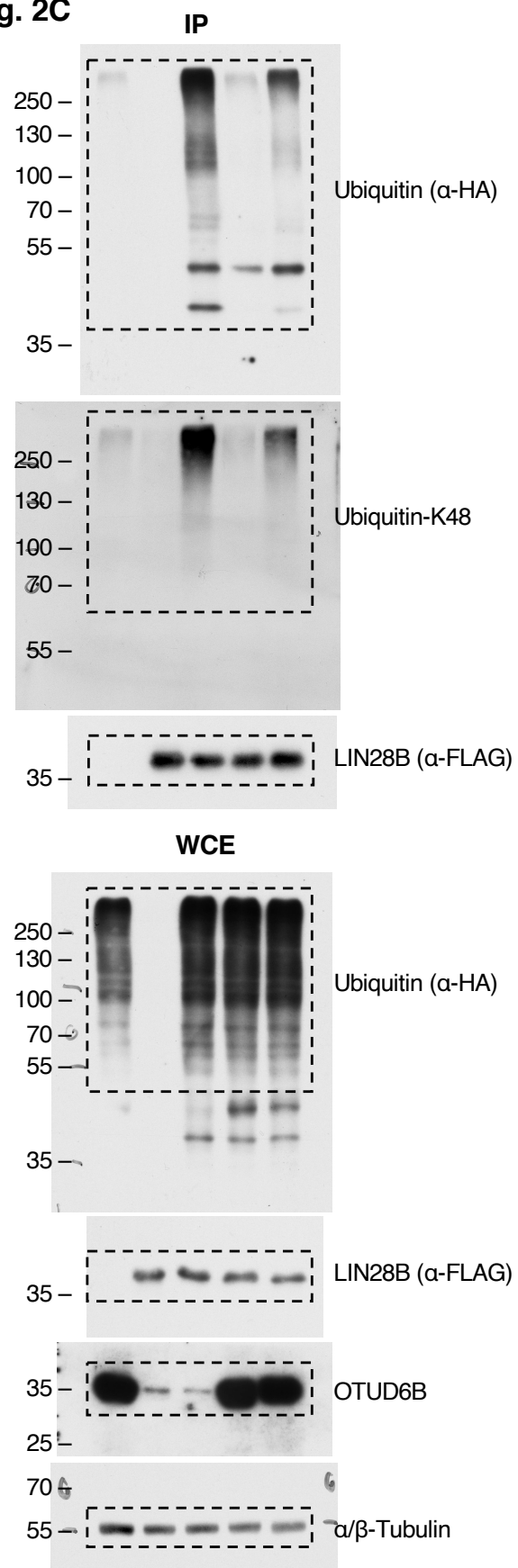

**Fig. 2D**

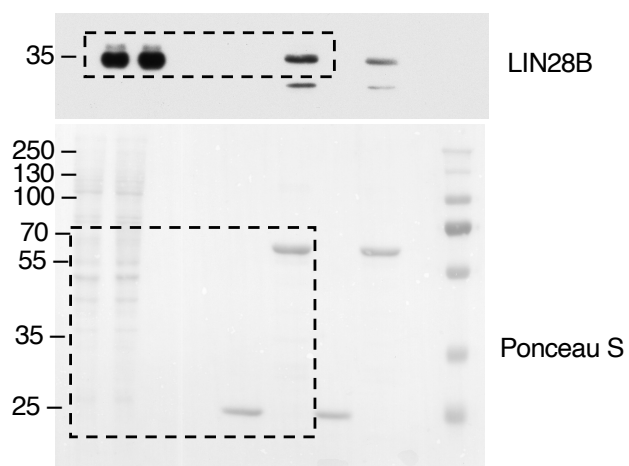

Fig. 2E

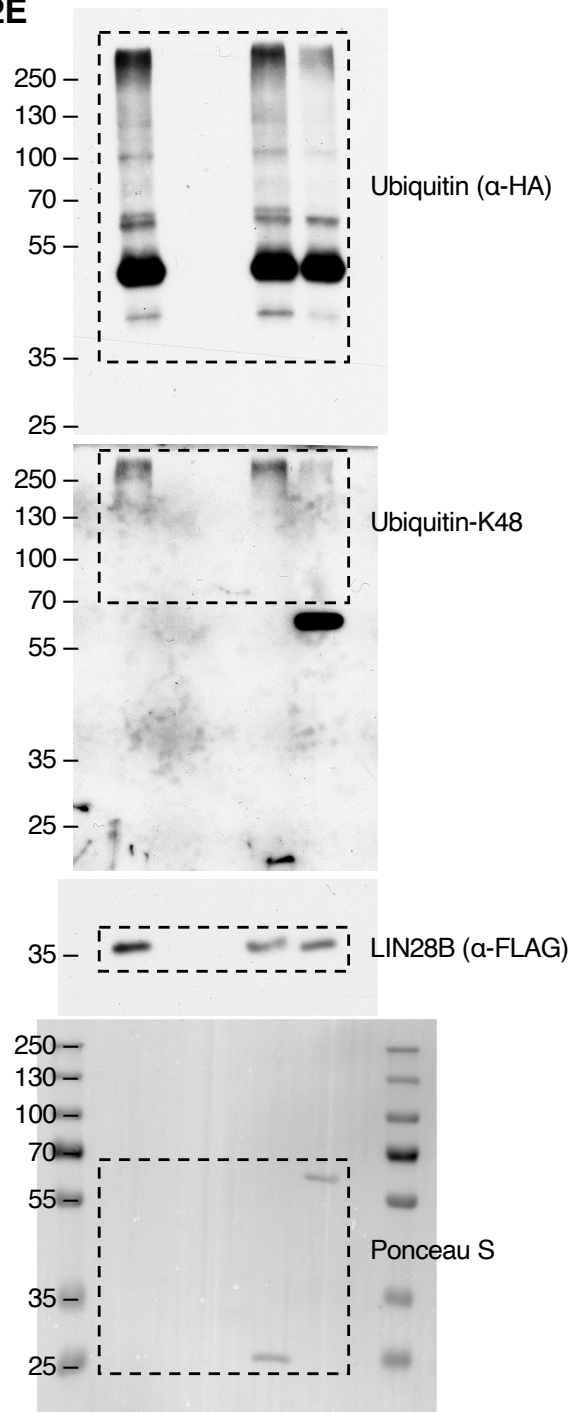

Supplement: Supplementary file 7 — Source Data for Figure 2 [file EMBJ-41-e110871-s008.pdf]

**Fig. 3A**

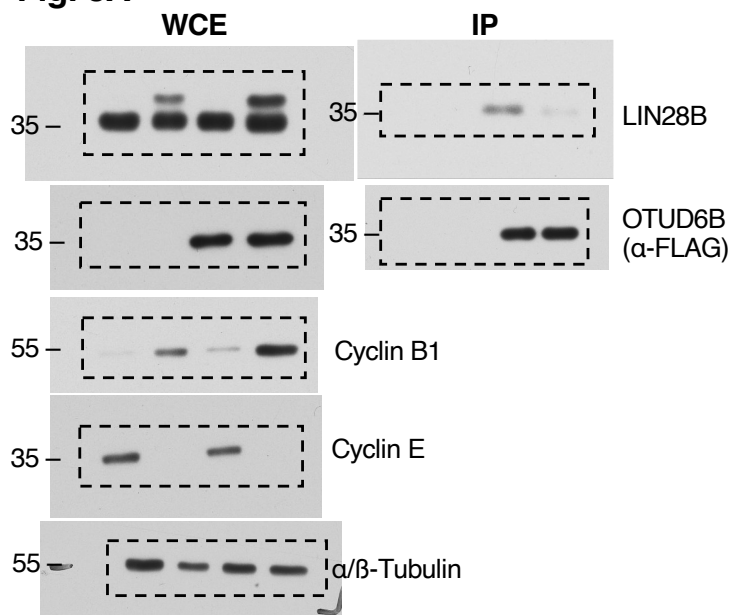

**Fig. 3C**

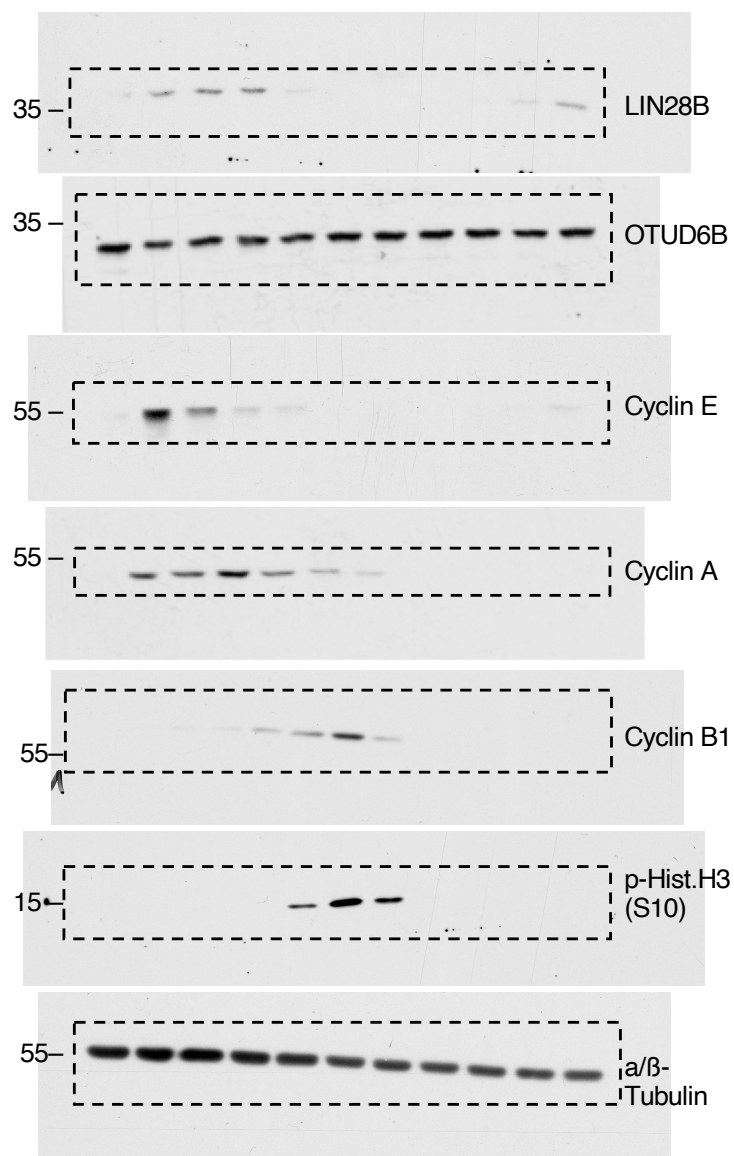

**Fig. 3B**

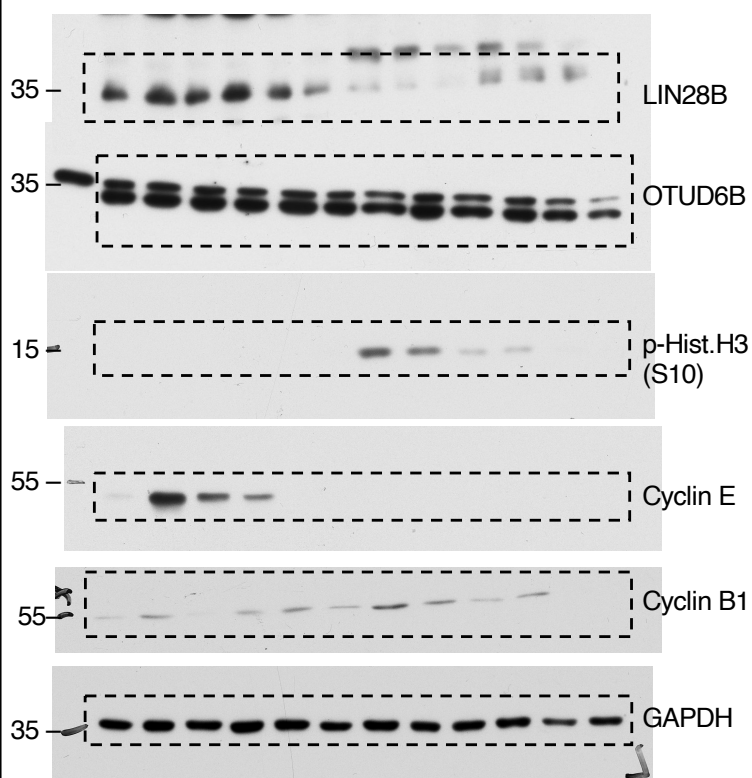

**Fig. 3D G1/S**

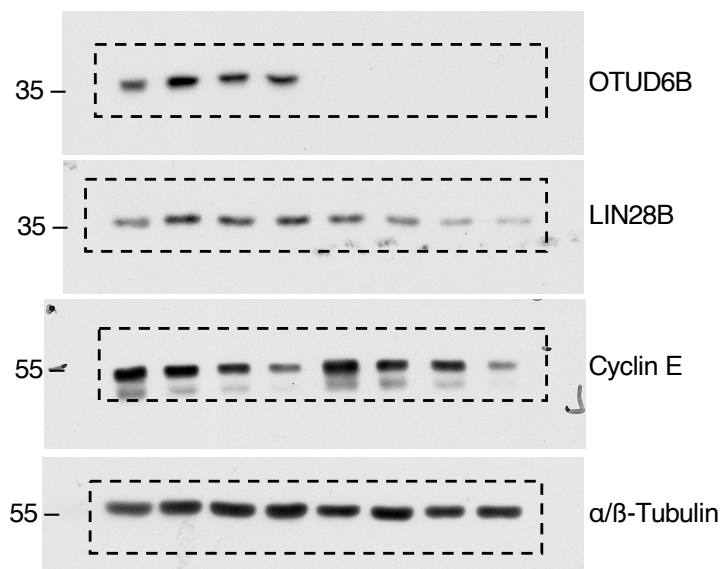

**Fig. 3D asynchronous**

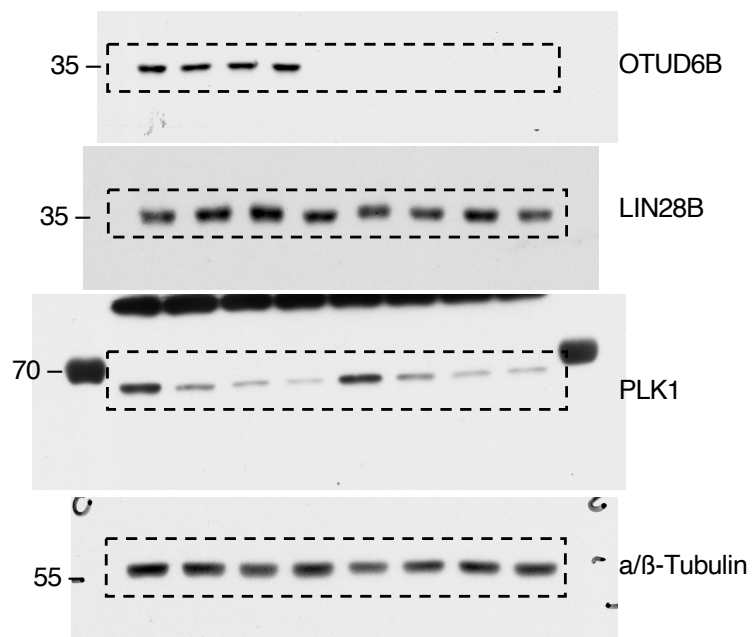

Supplement: Supplementary file 8 — Source Data for Figure 3 [file EMBJ-41-e110871-s003.pdf]

**Fig. 4D**

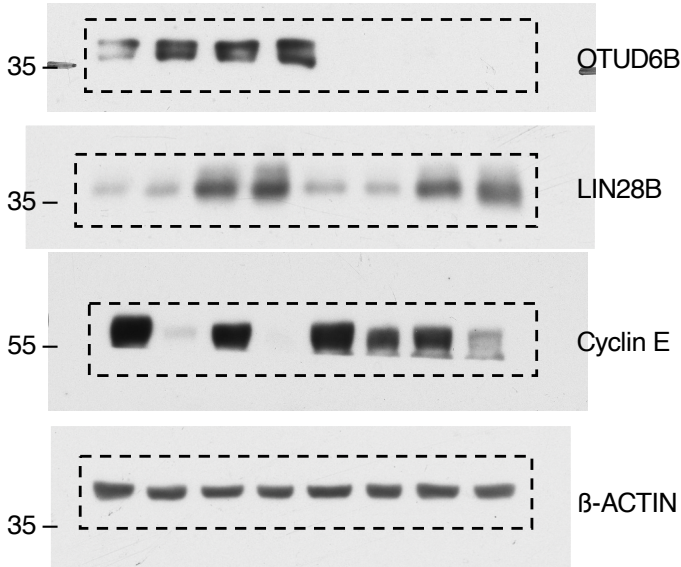

Supplement: Supplementary file 9 — Source Data for Figure 4 [file EMBJ-41-e110871-s007.zip › Source Data Fig. 4_uncropped.pdf]

**Fig. 5C**

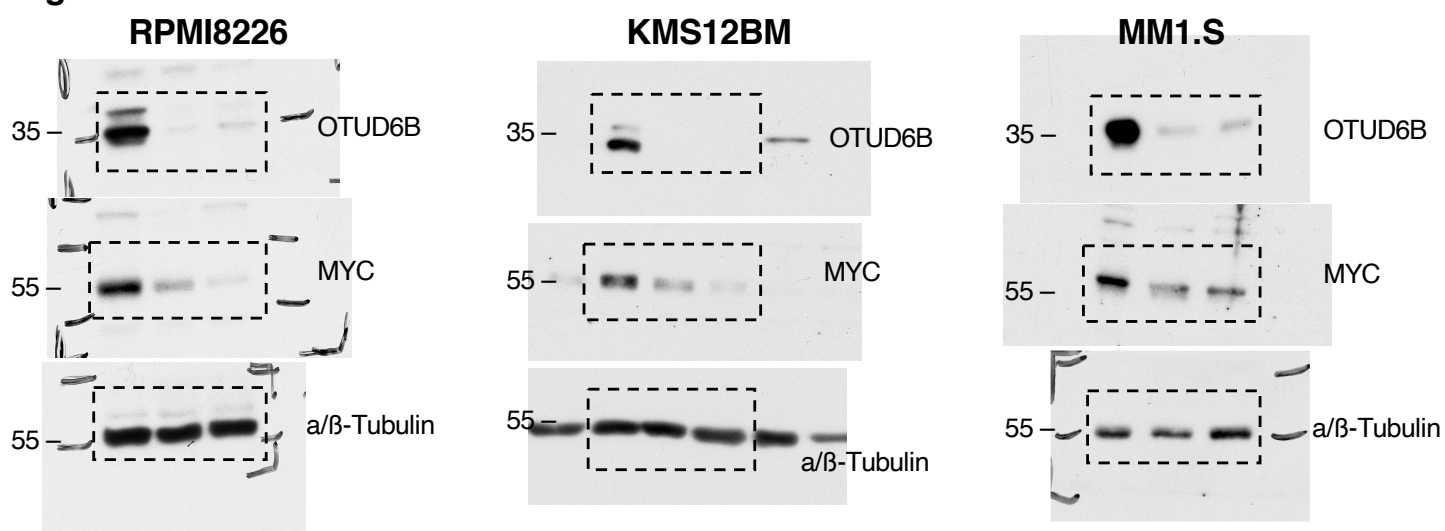

Supplement: Supplementary file 10 — Source Data for Figure 5 [file EMBJ-41-e110871-s010.zip › Source Data Fig. 5_uncropped.pdf]
