# Supplementary material for: The OTUD6B‐LIN28B‐MYC axis determines the proliferative state in multiple myeloma
Source: EMBO J. 2022 Sep 5;41(20):e110871. doi: 10.15252/embj.2022110871 (PMC9574752; doi:10.15252/embj.2022110871)

EV - Fig. 1C

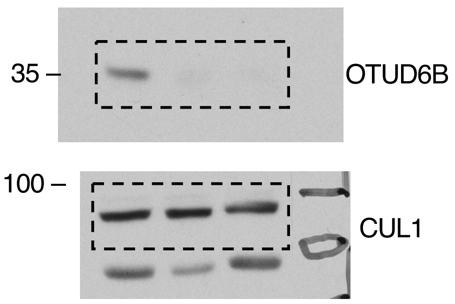

EV - Fig. 1E

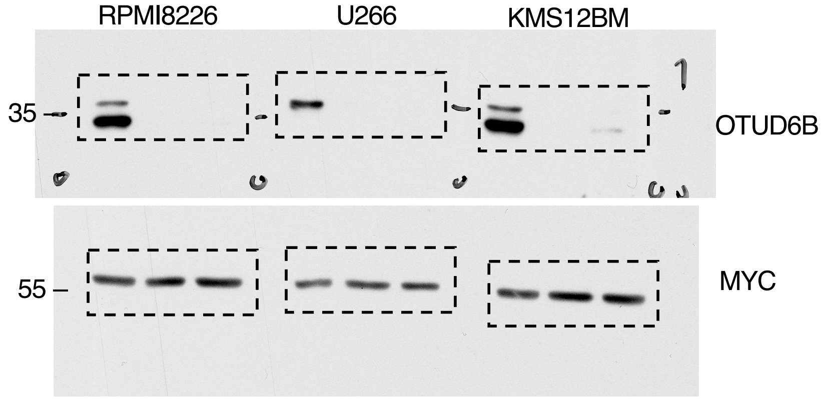

EV - Fig. 1G

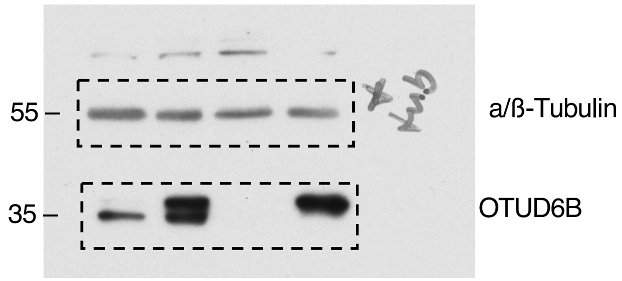

Supplement: Supplementary file 3 — Source Data for Expanded View and Appendix [file EMBJ-41-e110871-s011.zip › EV and Appendix Figures.zip!/EV Figures.zip!/Extended View Figure EV1/Source Data Extended View Fig. 1_uncropped.pdf]
