# Supplementary material for: The OTUD6B‐LIN28B‐MYC axis determines the proliferative state in multiple myeloma
Source: EMBO J. 2022 Sep 5;41(20):e110871. doi: 10.15252/embj.2022110871 (PMC9574752; doi:10.15252/embj.2022110871)
Supplement: Supplementary file 3 — Source Data for Expanded View and Appendix [file EMBJ-41-e110871-s011.zip › EV and Appendix Figures.zip!/EV Figures.zip!/Extended View Figure EV4/Source Data Extended View Fig. 4_uncropped.pdf]

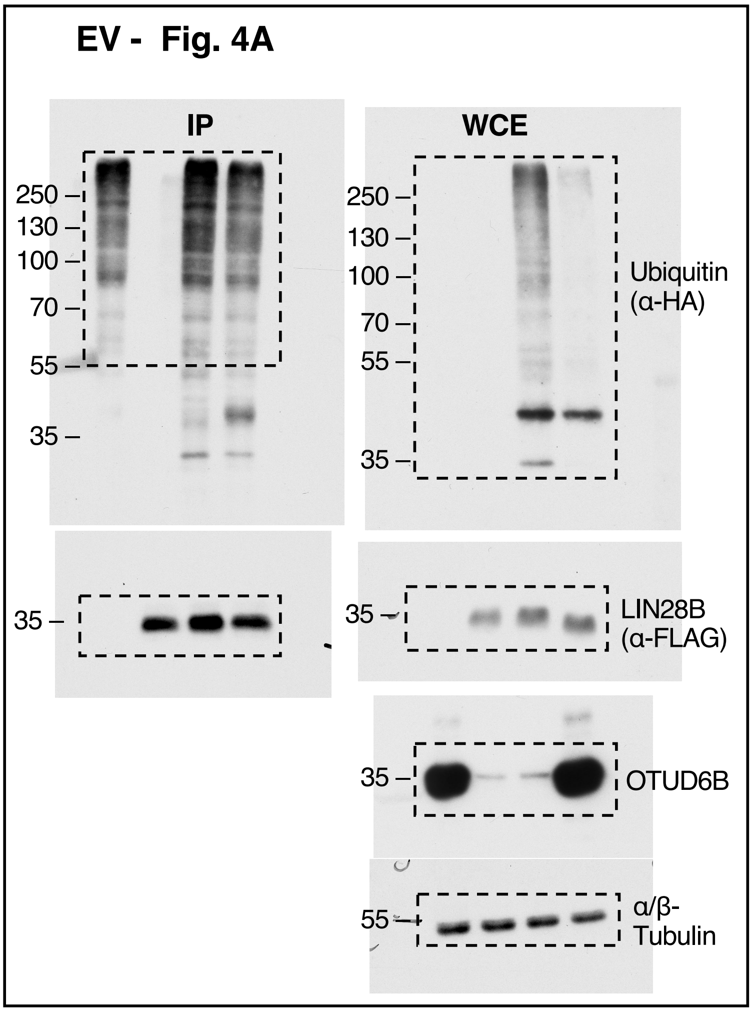
EV - Fig. 4A
IP
WCE
250
130
100
70
55
35
Ubiquitin
(α-HA)
LIN28B
(α-FLAG)
OTUD6B
α/β-
Tubulin

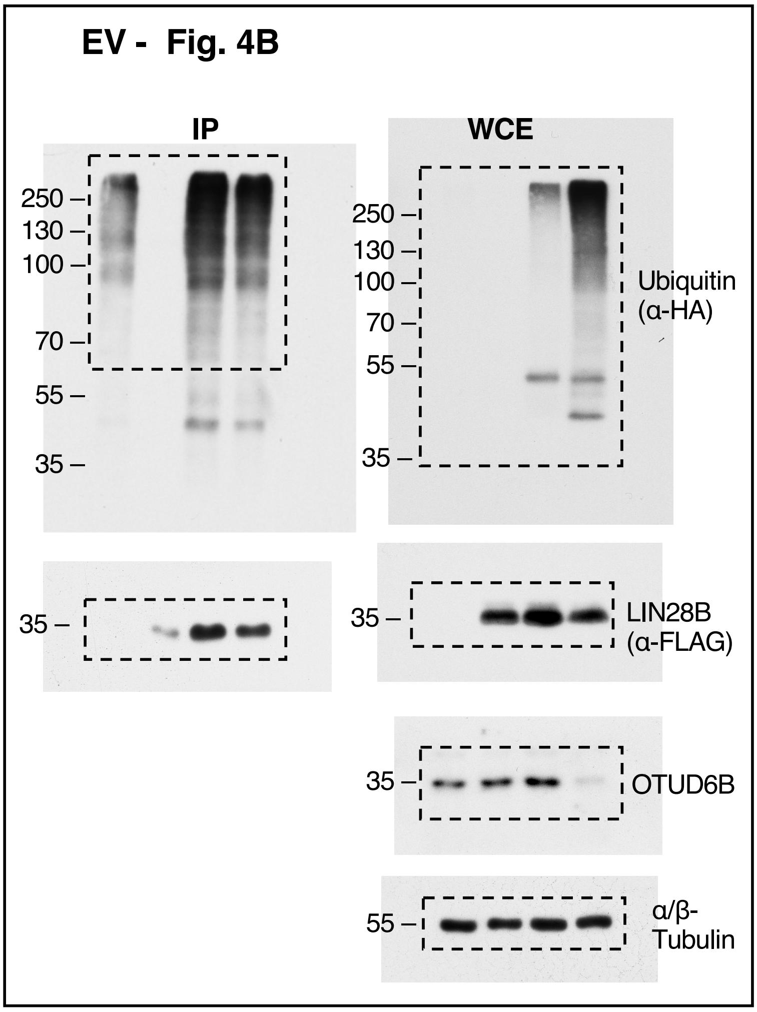
EV - Fig. 4B
IP
WCE
250
130
100
70
55
35
Ubiquitin
(α-HA)
LIN28B
(α-FLAG)
OTUD6B
α/β-
Tubulin
